# Death of Dr. Early W. Walton

**Published:** 1888-10

**Authors:** 


DEATH OF DR. WALTON.—Dr. Early W. Walton, formerly of Austin, died in New York very recently, his death causing a great shock and grief to a large circle of admiring and warmly attached friends, both here and in New York. He was a most accomplished young physician, and his death was untimely and deplorable; cut off, as it were, at the very threshold of a promising career. Graduating at Tulane University, he practiced a while in Williamson county, but choosing the eye and ear for a specialty, he took a general course at Bellevue, and graduated M. D. also from that famous institution. Two years ago, he attended a special course at the Manhattan Eye and Ear Infirmary, and was immediately made second assistant physician there. From this position he rapidly rose, by virtue of his fitness and qualification, professionally, combined with his beautiful social traits of character, to first assistant, and then to chief house surgeon. The latter position he was permitted to hold only a few weeks, when he was attacked with acute obstruction of the bowels, which necessitated laparotomy, and from the effects of which he died.

Dr. Walton was a native of Carroll county, Mississippi, and was a son of Col. W. M. Walton of Austin. He was a Christian gentleman, in the fullest sense, and a physician of a high order of attainments, as well as of natural endowments. His friends loved him; and when his remains reached Austin, a very large concourse of people, of all classes and conditions, followed them to their last resting place. Truly, "death loves a shining mark," and no nobler heart was ever stilled by the arrows of the "insatiate archer," than that of Dr. Early Walton. Our sympathy is extended to his bereaved parents, and we, with a host of friends, mourn his loss sincerely.

